# FOXO1 Is a Critical Switch Molecule for Autophagy and Apoptosis of Sow Endometrial Epithelial Cells Caused by Oxidative Stress

**DOI:** 10.1155/2021/1172273

**Published:** 2021-12-21

**Authors:** Jiayin Lu, Jiaqiang Huang, Shisu Zhao, Wenjiao Xu, Yaoxing Chen, Yuanyuan Li, Zixu Wang, Yanjun Dong, Renrong You, Jing Cao, Yulan Dong

**Affiliations:** ^1^College of Veterinary Medicine, China Agricultural University, Haidian, Beijing 100193, China; ^2^Key Laboratory of Precision Nutrition and Food Quality, Ministry of Education, Department of Nutrition and Health, China Agricultural University, Haidian, Beijing 100193, China

## Abstract

Oxidative stress (OS) is involved in various reproductive diseases and can induce autophagy and apoptosis, which determine the different fates of cells. However, the sequence and the switch mechanism between autophagy and apoptosis are unclear. Here, we reported that chronic restraint stress (CRS) induced OS (decreased T-AOC, T-SOD, CAT and GSH-Px and increased MDA) and then disturbed the endocrine environment of sows during early pregnancy, including the hypothalamic-pituitary-ovarian (HPO) and the hypothalamic-pituitary-adrenal (HPA) axes. Meanwhile, after CRS, the KEAP1/NRF2 pathway was inhibited and attenuated the antioxidative ability to cause OS of the endometrium. The norepinephrine (NE) triggered *β*_2_-AR to activate the FOXO1/NF-*κ*B pathway, which induced endometrial inflammation. CRS induced the caspase-dependent apoptosis pathway and caused MAP1LC3-II accumulation, SQSTM1/p62 degradation, and autophagosome formation to initiate autophagy. Furthermore, *in vitro*, a cellular OS model was established by adding hydrogen peroxide into cells. Low OS maintained the viability of endometrial epithelial cells by triggering autophagy, while high OS induced cell death by initiating caspase-dependent apoptosis. Autophagy preceded the occurrence of apoptosis, which depended on the subcellular localization of FOXO1. In the low OS group, FOXO1 was exported from the nucleus to be modified into Ac-FOXO1 and bound to ATG7 in the cytoplasm, which promoted autophagy to protect cells. In the high OS group, FOXO1 located in the nucleus to promote transcription of proapoptotic proteins and then induce apoptosis. Here, FOXO1, as a redox sensor switch, regulated the transformation of cell autophagy and apoptosis. In summary, the posttranslational modification of FOXO1 may become the target of OS treatment.

## 1. Introduction

Oxidative stress (OS) is widely present in various physiological and pathological phenomena in the process of life, including cancer [1], metabolism [2], preeclampsia [3], and ageing [4]. In modern society, humans are challenged not only by acute psychological stress but also by chronic psychological stress during their daily life [5]. Under acute oxidative stress (AOS), antioxidant systems are capable of adequately coping with enhanced reactive oxygen species (ROS) amounts, and the level returns to its initial status. In contrast, chronic oxidative stress (COS) can increase ROS levels, which can enhance the modification of different cellular components, substantially disturbing homeostasis. The lowest (mild) intensity OS is sensed by the nuclear factor-erythroid 2 (NF-E2)- related factor 2 (NRF2)/KEAP1 system, which is known to be activated by minute amounts of ROS. The cell cannot cope with highly intensive oxidative stress and may enter apoptosis [6]. However, the switch mechanism between AOS and COS remains unclear.

OS can induce autophagy, apoptosis, and inflammation [7–9]. Upon encountering OS, autophagy acts rapidly and effectively to remove oxidized proteins or organelles, including damaged mitochondria that generate more ROS, thereby indirectly contributing to the maintenance of redox homeostasis [10]. Meanwhile, endometrial autophagy is essential for the implantation of embryo, and it may be associated with the decidualization of endometrial during early pregnancy [11]. In eukaryotic cells, over 90% of ROS are produced by mitochondria [12], which is accompanied by energy production in the form of ATP. Apoptosis occurs when the excessive ROS are produced. Apoptosis is accompanied by a sequence of characteristic biochemical changes, including mitochondrial outer membrane permeabilization (MOMP); activation of the effectors, CASP3, CASP6 and CASP7; and the activation of catabolic hydrolases that degrade most of the macromolecules of the cell, which includes DNA [13]. Autophagy has been found to be closely related to apoptosis [14]. Autophagy and apoptosis often occur in the same cell, mostly in a sequence in which autophagy precedes apoptosis [15]. Again, it seems plausible that the cytoprotective autophagy is triggered by low levels of stress, whereas more intense and protracted stress culminates in apoptotic demise [16]. However, the switch mechanism of autophagy and apoptosis remains unclear.

Forkhead box proteins (FOXOs) are family of transcription factors that play important roles in the regulation of genes involved in cell growth, proliferation, differentiation, apoptosis, inflammation, and longevity. There are four FOXO family members in humans, FOXO1, FOXO3, FOXO4, and FOXO6 [17, 18]. Cholesterol (CHO) triggers ROS generation and activates the AKT/FOXO1 pathway [19]. Mammalian sterile20-like kinase 1 (MST1) is activated by ROS produced in mitochondria in response to hypoxia and activated MST1 promotes the nuclear import of FOXO1 [20]. Therefore, FOXOs act as the cellular redox sensors that become modified posttranslationally through phosphorylation, acetylation, and ubiquitination in response to OS [21]. Posttranslational modification of FOXO1 is an important mechanism that regulates its ability to activate distinct gene sets involved in cell cycle arrest, apoptosis, OS, and DNA repair [22]. The binding of FOXO1 acetylation to ATG7 promotes autophagy for cell survival to resist OS [23]. FOXO1 in the nucleus increases the translation of B-cell lymphoma-2-like protein 1(BIM) to promote apoptosis [24, 25]. The levels of ROS affect FOXO cytoplasmic/nuclear shuttling, FOXO stability, and eventually transcription of FOXO-controlled target genes [21]. However, it is not clear whether the subcellular localization of FOXO1 is related to autophagy and apoptosis caused by different levels of OS.

Here, a restraint stress model was used to explore the switch mechanism between autophagy and apoptosis caused by OS duration. The model is based on the individual sow stalls, which is a global problem in the breeding industry. Furthermore, porcine endometrial epithelium cells (PEECs) were used to explore the mechanism of FOXO1 in autophagy and apoptosis under OS. FOXO1 was the switch factor of autophagy and apoptosis caused by OS, namely, FOXO1 promoted autophagy in the cytosol and facilitated apoptosis in the nucleus.

## 2. Materials and Methods

### 2.1. Animal Treatment

**Figure 1:**
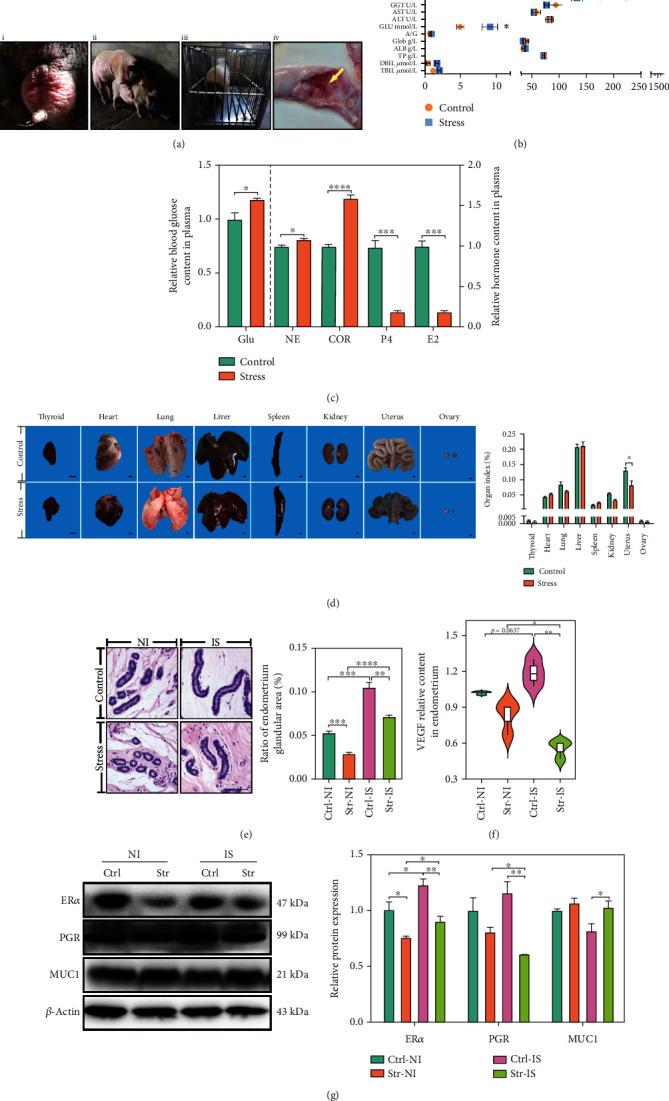
Chronic restraint stress (CRS) prevented the development of the endometrium by disrupting HPA axes and HPO axes. (a) Representative images of oestrus (i), mating (ii), restraint stress model (iii), and uterus implantation site (yellow arrow) (iv) of sow. (b) All sows were offered an indepth health assessment, including routine blood test (Figure S1C) and blood biochemistry assay. See the abbreviation list for the full name of abbreviations. (c) Blood glucose of ear vein was detected. The COR, P4, and E2 concentration levels in plasma were measured via RIA. (d) Representative images and quantification of organs in CRS and control group of early pregnancy sow. Bar :1 cm. (e) Representative images of H&E staining of sow endometrium and quantification of ratio of endometrium gland area. Bar: 100 *μ*m. (f) ELISA analysis of VEGF protein levels in endometrium of early pregnancy sow. (g) Immunoblotting analysis of ER*α*, PGR, and MUC1 protein levels in endometrium of early pregnancy sow. Glu: glucose; COR: cortisol; NE: norepinephrine; P4: progesterone; E2: estradiol; VEGF: vascular endothelial growth factor. IS: implantation site; NI: nonimplantation site; Str: stress; Ctrl: control. *N* = 4; ^∗^*p* < 0.05, ^∗∗^*p* < 0.01, ^∗∗∗^*p* < 0.001, ^∗∗∗∗^*p* < 0.0001. ns: no significant.

Eight Bama minisows (25-35 kg), purchased from Beijing Shichuang Century minipig Breeding Base, were raised in a normal environment. Oestrus was observed (Figure 1(a)(i), and the sows mated naturally (Figure 1(a)(ii). On the first day of gestation, four of the sows were placed in the sow individual stalls (SIS, 100 cm × 50 cm × 60 cm; they had free access to food and water, but could not turn around), and the other four were raised in group housing (Figure 1(a)(iii). On the 18th day of pregnancy (mid-implantation), anesthesia was performed, and blood was collected. Organs were photographed, and endometrial tissues at the implantation site (IS) (Figure 1(a)(iv) and nonimplantation site (NI) were collected and stored in liquid nitrogen, while the uterus of the implantation site and the nonimplantation site was preserved in 4% paraformaldehyde (PFA) and 2.5% glutaraldehyde. Three implantation sites and nonimplantations were selected in the research. All sow procedures were performed in accordance with the guidelines of the China Agricultural University Institutional Animal Care and Use Committee (AW18079102-2-2). Ctrl-NI was a nonimplantation site, and Ctrl-IS was an implantation site in the control group. Str-NI was the nonimplantation site, and Str-IS was the implantation site in the CRS group.

### 2.2. Cell Culture and Treatment

The carotid artery of Bama mini sow was bled to death. Then, the uterine tissue was collected and placed in PBS containing penicillin-streptomycin. The connective tissue around the uterus was removed and washed three times with PBS. Subsequently, the tissue was transferred to 75% alcohol, soaked for 2 min, and then washed with PBS. The uterus was cut along the longitudinal axis to expose the uterine lumina and separate the endometrium with ophthalmic scissors and tweezers. The endometrium was rinsed in PBS until the liquid was clear.

The endometrial tissue was digested with three times its volume of 0.1% collagenase I (17018029, Gibco, USA) in a 37°C water bath for 1 hour (h). The digested tissue mixture was passed through a 100 *μ*m sieve, and the filtrate was collected and then centrifuged at 1,500 rpm for 5 min. The supernatant was discarded, and the precipitate was resuspended in Epithelial Cell Medium-animal (EpiCM-a, 4131, ScienCell). Meanwhile, the cells were identified by anti-CK19 antibody (Figure S1A). Subsequently, the cells were immortalized by infection with lentivirus expressing SV40T. The cells were regularly authenticated by an EZ-PCR *Mycoplasma* Detection Kit (20-700-20, Biological Industries, Israel), tested for the absence of *Mycoplasma* contamination and used within 5 passages after thawing (Figure S1B).

H_2_O_2_ was the regular drug to establish the OS model. Therefore, in our study, H_2_O_2_ was added to explore the duration and intensity of OS exposure. H_2_O_2_ was added at concentrations of 50 *μ*M, 100 *μ*M, 200 *μ*M, 400 *μ*M, 800 *μ*M, and 1000 *μ*M for 0.5 h, 1 h, 2 h, 3 h, 6 h, 12 h, and 24 h, respectively. Cell activity was detected via Cell Counting Kit-8 (CCK8, C0038, Beyotime Biotechnology, China). Briefly, cells were seeded and cultured in 96-well plates. After treatment, cells were washed with PBS twice and incubated with 100 *μ*L medium containing 10% CCK-8 solution at 37°C for 2 h in each well. The absorbance was measured at 450 nm using an automatic microplate reader (800TS, BioTek, USA). Then, the concentration was chosen. Leptomycin B (20 nM, LMB, 9676, Cell Signaling Technology, USA) was incubated for 3 h to inhibit the export of FOXO1 from the nucleus. Ivermectin (25 *μ*M, IVE, HY-15310, Med Chem Express, USA) was incubated for 1 h, which inhibited Imp*α*/*β*1 binding to FOXO1 and then prevented the import of FOXO1 to the nucleus. *In vitro*, slides of PEECs were used for morphological studies, and the SDS–PAGE electrophoresis was performed after protein extraction. Nuclear-cytoplasm separation experiments were performed according to the manufacturer's instructions for the Nuclear and Cytoplasmic Protein Extraction Kit (P0027, Beyotime Biotechnology, China).

### 2.3. Blood Index Detection Method

The blood was collected in the heart. Routine blood examination indices were detected via a blood cell analyser (MEK-7222, Japan) in whole blood. Blood biochemistry indices were determined by a blood biochemical analyser (TBA-120FR, Japan) in the serum. The blood glucose of the ear vein was detected by a Roche blood glucose meter (ACCU-CHEK Active, Roche Diagnostics GmbH, Germany). The levels of cortisol (COR) (D10TB), P4 (B08TB), and E2 (B05TB) were measured spectrophotometrically in diluted plasma using a radioimmunoassay kit according to the manufacturer's instructions. These kits were purchased from Beijing North Institute of Biotechnology.

### 2.4. Enzyme-Linked Immunosorbent Assay (ELISA)

The NE level of plasma was detected by a porcine norepinephrine ELISA Kit (CK-E95740, Beijing Laibotairui Technology Co., Ltd., China). In the endometrium, the levels of cytokines were measured via vascular endothelial growth factor (VEGF) (ELP-VEGF), interleukin-1*β* (IL-1*β*) (ELP-IL1b), tumour necrosis factor-*α* (TNF-*α*) (ELP-TNFa), interferon-*γ* (IFN-*γ*) (ELP-IFNg), interleukin-4 (IL-4) (ELP-IL4), and interleukin-13 (IL-13) (ELP-IL13) enzyme-linked immunosorbent assay (ELISA) kits according to the manufacturer's instructions. These ELISA kits were purchased from RayBiotech (USA).

### 2.5. Oxidative Stress Detection

The biomarkers of OS level include T-AOC (total antioxidant capacity), T-SOD (total superoxide dismutase), GSH-Px (glutathione peroxidase), CAT (catalase), and MDA (malondialdehyde). The antioxidative ability of plasma and endometrium was measured via the Total Antioxidant Capacity Assay Kit with the FRAP method (T-AOC, S0116), Total Superoxide Dismutase Assay Kit with WST-8 (T-SOD, S0101), Glutathione Peroxidase Assay Kit with NADPH (GSH-Px, S0056), Catalase Assay kit (CAT, S0051), and Lipid Peroxidation MDA Assay kit (MDA, S0131) with spectrophotometry. These kits were purchased from Beyotime Biotechnology (China).

### 2.6. Quantitative RT–PCR (qRT–PCR)

Total RNA from the endometrium was extracted using the TRIzol Reagent (CW0580, CWBIO, China) and reverse transcribed into cDNA using the GoScript™ Reverse Transcription System (A5001, Promega, USA). Real-time PCR was performed using the AceQ qPCR SYBR Green Master Mix (Q121-02, Vazyme, China) with the ABI Step-One Plus™ Real-time PCR system (Applied Biosystems, Foster City, CA, USA). The data were analysed using the 2^−*Δ*Ct^ method and calculated using UBB as the internal reference. The primer sequences are presented in *Supplementary Table*1.

### 2.7. Hematoxylin and Eosin (HE) Staining

Uterine tissues were immediately fixed in 4% PFA in 0.1 M phosphate-buffered saline (pH 7.4) for one week and embedded in paraffin for sectioning (5 *μ*m). These tissue sections were stained with haematoxylin and eosin. At least 45 random fields in five sections of each sample were photographed with a microscope (BX51, Olympus, Tokyo, Japan). The gland area and endometrial area were measured via Image-Pro Plus (Media Cybernetics, USA). Meanwhile, the ratio of gland area to endometrium area was calculated.

### 2.8. Immunohistochemistry (IHC) Staining

The sections experience dewaxing followed by antigen retrieval in 0.01 M citrate buffer and 3% hydrogen peroxide block. Subsequently, the sections were blocked with 5% goat serum for 30 min at 37°C and then incubated with antibodies (*Supplementary Table*2) or goat serum (negative control) overnight at 4°C. The next day, the secondary antibodies [1 : 300, goat anti-rabbit IgG (H + L), biotin-conjugated, CW0107S, CWBIO, China] were incubated for 2 h at room temperature. After washing, the tissues were incubated with streptavidin-horseradish peroxidase (1 : 300, SA-5004, Vector Laboratories) for 2 h at room temperature. Immunoreactivity was visualized by incubating the tissue sections with a DAB staining kit (ZLI-9017, ZSGB-BIO) and counterstaining with haematoxylin. Immunoreactive cells were stained yellow-brown. The positive cells were counted in 25 random fields from five cross-sections in each sample. The mean integral optical density (IOD) of the positive cells was determined by Image-Pro Plus 6.0 (Media Cybernetics, USA).

### 2.9. Immunofluorescence Staining

The sections were blocked with 5% goat serum for 30 min at 37°C. Then, the sections were incubated with diluted primary anti-LC3B and anti-SQSTM1/p62 antibodies, and coverslips of PEECs were incubated with anti-FOXO1 (*Supplementary Table*2) overnight at 4°C. After incubation, coverslips and sections were washed with PBS three times and incubated with goat anti-rabbit IgG conjugated with Alexa Fluor 594 (red for FOXO1 and LC3B, 1 : 300, #8889, Cell Signaling Technology, USA) or goat anti-mouse conjugated with Alexa Fluor 488 (green for SQSTM1/p62, 1 : 300, #4408, Cell Signaling Technology, USA) secondary antibody for 2 h in the dark at room temperature. Subsequently, the cell nucleus was counterstained with DAPI (C0065, Solarbio, China) for 10 min. Imaging was performed on a fluorescence microscope (BX51, Olympus, Tokyo, Japan) and measured using Image-Pro Plus 6.0.

### 2.10. Transmission Electron Microscopy (TEM)

The tissue fixed in glutaraldehyde was washed with PBS and fixed with 1% osmium acid for 2 h. After gradient alcohol dehydration, the alcohol was replaced with anhydrous acetone and immersed in a mixture of embedding agent and acetone in a 35°C incubator overnight. The tissue was exposed and sliced and stained with uranium acetate and lead citrate. The cells were observed and photographed under a transmission electron microscope (Hitachi H-7500, Tokyo, Japan).

### 2.11. MDC Staining

Cells were washed twice with a wash buffer. Then, 100 *μ*L of the dansylcadaverine (MDC) was added to the well and incubated in the dark for 45 min at room temperature. After three washes with the buffer, 100 *μ*L of collection buffer was added, and the cells were observed under a fluorescence microscope (excitation filter wavelength 355 nm, blocking filter wavelength 512 nm). Then, the positive cells in 100 cells were counted and photographed by fluorescence microscopy (BX51, Olympus, Tokyo, Japan).

### 2.12. Flow Cytometry to Detect Cell Apoptosis

To quantify the cell apoptotic rate, cells were digested with 0.25% trypsin solution without EDTA after washing in cold PBS. Proteolysis was neutralized with FBS, and the lysates were centrifuged at 3,000 rpm for 5 min, washed once with PBS, and stained using the Annexin V-FITC and a propidium iodide (PI) solution (C1062M, Beyotime Biotechnology, China) for 15 min at room temperature away from light. The percentage of apoptotic cells for each sample was subsequently evaluated by a BD FACSCalibur flow cytometer (BD Biosciences, San Jose, USA).

### 2.13. Western Blot Analysis

Cells (washed three times with PBS) and the tissues were lysed in a RIPA lysis buffer (CW2333, CWBIO, China) with protease inhibitor (CW2200, CWBIO, China) and phosphatase inhibitor cocktail (CW2383, CWBIO, China) for 45 min. Protein concentrations of cell lysates and tissue lysates were determined using a Pierce BCA Protein Assay Kit (CW0014, CWBIO, China) according to the manufacturer's instructions. Total protein (30-50 *μ*g) for each sample was separated by the SDS–PAGE and transferred to polyvinylidene difluoride (PVDF, Millipore, IPVH00010) membranes. The membranes were blocked with 5% nonfat milk at room temperature for 2 h and then incubated with primary antibodies overnight at 4°C (for all antibodies, see *Supplementary Table*2). After washing, the membranes were incubated with a 1 : 8,000 dilution of goat anti-mouse or anti-rabbit horseradish peroxidase (HRP)-conjugated secondary antibodies (CW0102 and CW0103, CWBIO, China) at room temperature for 1.5 h. Protein bands were visualized using an Immobilon Western Chemiluminescent HRP Substrate (WBKLS0500, Millipore) via an automatic chemiluminescence imaging analysis system (Tanon-5200, USA). ImageJ (NIH, National Institutes of Health, USA) was used to analyse these images.

### 2.14. Immunoprecipitation and Immunoblot Analysis

Total protein from whole-cell lysate was immunoprecipitated. The extract was incubated by rotating for 3 h in 4°C with protein A/G beads (sc-2003, Santa Cruz, USA). The incubated beads were discarded to clear the nonspecific protein, and 20 *μ*L of the supernatant was taken as input. Then, ATG7 antibody (1 : 500, 8558, Cell Signaling Technology, USA) was added and incubated with rotation for 12 h at 4°C. The beads were centrifuged at 3,000 rpm for 5 min and washed rotating with RIPA lysis buffer (P0013C, Beyotime Biotechnology, China). DTT (ST041, Beyotime Biotechnology, China) was added into the buffer. The beads were resuspended in SDS–PAGE loading buffer (CW0027, CWBIO, China) and then incubated at 99°C for 10 min. The SDS–PAGE electrophoresis test was performed after protein extraction. The immunoreactive bands were incubated with Ac-FOXO1 antibody overnight at 4°C. Protein bands were visualized via an automatic chemiluminescence imaging analysis system (Tanon-5200, USA).

### 2.15. Statistical Analysis

All animal experiments were performed in quadruplicate, and cell experiments were conducted at least three times. Data were shown as the mean ± SEM. Statistical analysis was performed using the GraphPad Prism software 8.3.2 using two-tailed Student's *t*-test with 95% confidence limits. ^∗^*p* < 0.05; ^∗∗^*p* < 0.01; ^∗∗∗^*p* < 0.001, and ^∗∗∗∗^*p* < 0.0001 were considered significan,. ns: no significant. The effects of implantation, stress, and their interaction on the indexes were analyzed by the two-way ANOVA (Table S3). In the graph containing multiple indicators, the control group was used as the standard for normalization. Violin diagrams were made in the R 4.0.1 via ggplot2. The code is:

ggplot(mydata, aes(Class, Value)) + geom_violin(aes(fill = Class), trim = FALSE) + geom_boxplot(width = 0.2) + scale_fill_manual(values = c(brewer.pal(7, ^"^Set2^”^)[c(1,2,4,5)]))+ theme_light().

## 3. Results

### 3.1. CRS Disturbed the Development of the Sow Endometrium in Early Pregnancy via the HPA and HPO Axes

Using an *in vivo* CRS model target in pregnant sows (Figure 1(a)), we examined routine blood indices and blood biochemistry to observe the damage caused by CRS in pregnant sows. From the results of routine blood examination, the CRS group was not significantly different from the control group (Figure S1C). However, the blood biochemistry showed that the glucose was significantly increased in the CRS group compared with the control group (Figure 1(b)). Therefore, this study speculated that the CRS group did not induce a significant systemic inflammatory response but may have induced a stress response. To verify this hypothesis, the blood glucose of the ear vein was measured via a Roche blood glucose metre, and the blood glucose of the CRS group was increased compared with that of the control group (Figure 1(c)). Stress hormones, including COR and NE, which are markers in the stress process [26], were detected in plasma via RIA and ELISA, respectively. The COR levels in the CRS group were increased compared with those in the control group (Figure 1(c)), and the NE was increased compared with that in the control group (Figure 1(c)). The obtained results showed that the CRS model successfully induced a stress response in this work through the hypothalamic-pituitary-adrenal (HPA) axes.

Of note, the CRS did not cause any visible lesions in various organs (Figure 1(d)), while the uterine index was decreased in the CRS group (Figure 1(d)). CRS decreased the number of fetal but not was significant (Figure S2). The development of endometrial glands was affected after CRS [27]. In our work, the ratio of endometrial glandular area (REGA) in the IS group was increased compared with that in the NI group in both the control group and the CRS group. However, after CRS, the REGA was decreased in the NI uterus and in the IS of uterus compared with the control group (Figure 1(e)). In the uterus, VEGF plays a critical role in the development of uterine blood vessels. It was well demonstrated that the result of VEGF level was the same as the REGA level in the control group. CRS reduced VEGF levels in the IS and NI (Figure 1(f)). Thus, CRS blocks the development of the endometrium and blood vessel formation.

Progesterone (P4) and oestradiol (E2) are required for the implantation and pregnancy processes. The data of P4 and E2 by RIA suggested that they were downregulated in the CRS group (Figure 1(c)). Meanwhile, the receptor proteins of E2 and P4, called ER*α* and PGR, are signaling responses to external signals. The ER*α* protein levels in IS were increased compared with the NI, while the ER*α* protein levels in CRS were decreased compared with the control group (Figure 1(g)). In CRS, the PGR protein level in IS was decreased compared with NI. The PGR protein level in the CRS group was decreased compared with that in the control group (Figure 1(g)). Increased MUC1 is harmful to the implantation [28]. The MUC1 protein in IS was decreased compared to the NI. However, after CRS, the MUC1 protein was upregulated in the IS compared with the control (Figure 1(g)). Therefore, the development of the endometrium was affected by the hypothalamic-pituitary-ovary (HPO) axis.

Overall, CRS weakens the development of the endometrium in the early pregnancy. This process is triggered by COR and NE, which inhibit P4 and E2 in plasma. Blood vessel formation was blocked, and CRS prevented implantation by enhancing MUC1 expression.

### 3.2. CRS Induced OS and Inflammation via the *β*_2_-AR/FOXO1 and NF-*κ*B Pathways

**Figure 2:**
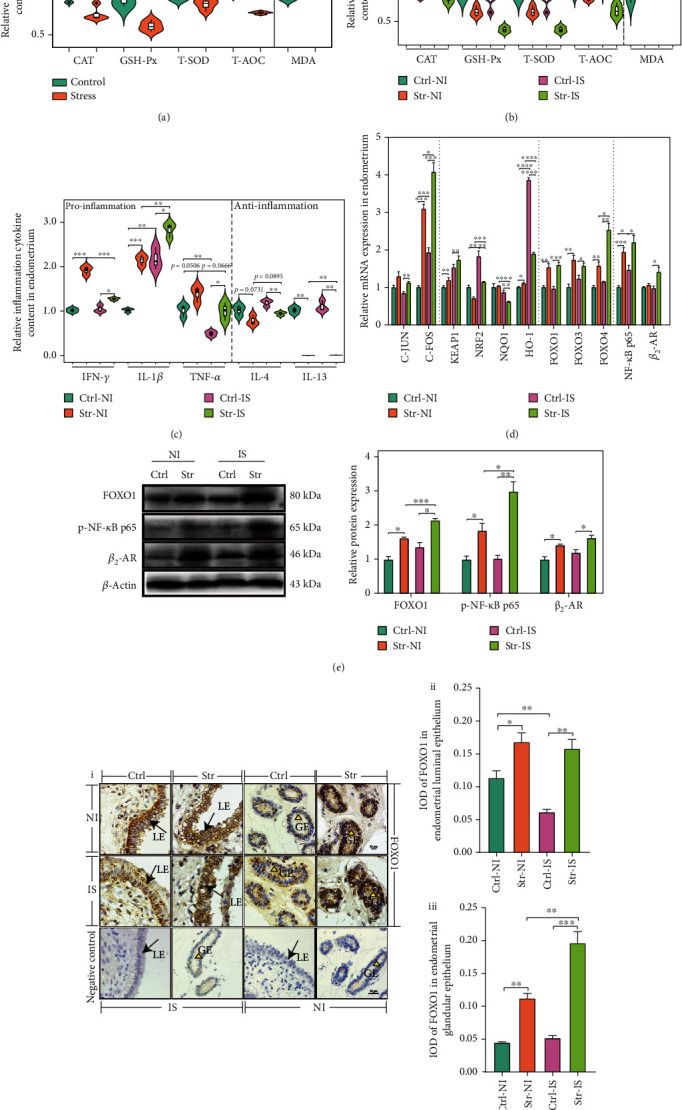
CRS enhanced the oxidative stress (OS) and inflammation by *β*_2_-AR pathway and FOXO1/NF-*κ*B p65 pathway. (a) The analysis of antioxidative enzyme (CAT, GSH-Px, T-SOD, and T-AOC) and lipid peroxide (MDA) in plasma. (b) The analysis of antioxidative enzyme (CAT, GSH-Px, T-SOD, and T-AOC) and lipid peroxide (MDA) in endometrium. (c) The analysis of proinflammatory cytokines (IFN-*γ*, IL-1*β*, and TNF-*α*) and anti-inflammatory (IL-4 and IL-13) cytokines in endometrium. (d) qRT-PCR was performed to measure the mRNA levels of *AP-1* (*C-JUN* and *C-FOS*), antioxidative-related genes (*KEAP1*, *NRF2*, *NQO1* and *HO-1*), *FOXO* family genes (*FOXO1*, *FOXO3* and *FOXO4*), proinflammation-related gene (*NF-κB p65*), and adrenergic receptor (*β_2_-AR*). (e) After CRS, the protein level of FOXO1, *β*_2_-AR, and phosphorylation NF-*κ*B p65 in endometrium was determined by Western blotting. (f) i, Immunohistochemical staining of FOXO1 on luminal epithelium (LE) (the black arrow) and glandular epithelium (GE) (the yellow triangle) in endometrium. Bar: 20 *μ*m. Semiquantitative analysis of IOD of FOXO1 in endometrial luminal epithelium (ELE) (ii) and endometrial glandular epithelium (EGE) (iii). CAT: catalase; GSH-Px: glutathione peroxidase; T-SOD: total superoxide dismutase; T-AOC: total antioxidant capacity; MDA: malondialdehyde; IFN-*γ*: interferon gamma; TNF-*α*: tumor necrosis factor alpha; IL-1*β*: interleukin 1 beta; IL-4: interleukin 4; IL-13: interleukin 13; FOXO1: forkhead box o 1; FOXO3: forkhead box o 3; FOXO4: forkhead box o 4; NF-*κ*B p65: nuclear factor kappa B p65; *β*_2_-AR: beta 2-adrenergic receptor; IS: implantation site; NI: nonimplantation site; Str: stress; Ctrl: control. *N* = 4, ^∗^*p* < 0.05, ^∗∗^*p* < 0.01, ^∗∗∗^*p* < 0.001, ^∗∗∗∗^*p* < 0.0001. ns: no significant.

Next, we determined whether OS and inflammation were induced by CRS. In the plasma, we observed that antioxidative enzymes, including the CAT, GSH-Px, T-SOD, and T-AOC, were decreased in the CRS group compared with those in the control group. However, the MDA level was significantly increased in the CRS group (Figure 2(a)). In endometrial tissue, the CAT, GSH-Px, T-SOD, and T-AOC were clearly decreased compared with those in the control group, while the MDA in CRS was significantly increased in the IS and NI under CRS. Within the control group, the GSH-Px in the IS group was decreased compared with that in the NI group. Also, within the CRS group, the GSH-Px, T-SOD, and T-AOC in the IS group were significantly decreased compared with those in the NI group (Figure 2(b)). Together, the antioxidative ability of IS was more vulnerable to CRS.

OS can initiate inflammation. From Figure S1C, we know that the CRS did not cause systemic inflammation. Therefore, whether endometrial tissue undergoes inflammation was considered. Our results demonstrated that the proinflammatory cytokines (the IFN-*γ*, IL-1*β* and TNF-*α*) in the CRS group were increased compared with those in the control group (Figure 2(c)). In the CRS, the IFN-*γ* and TNF-*α* in IS were decreased compared with the NI group, while the IL-1*β* in the IS was increased both in the CRS and control group. The IL-1*β* is required for the pregnancy recognition process [29]. In the control group, the TNF-*α* in the IS group was decreased compared with that in the NI group (Figure 2(c)). Overall, the increase of IL-1*β* in the endometrium is beneficial for implantation. However, it is harmful to the development of endometrium when it is increased abnormally. Additionally, in the IS and NI groups, the IL-4 and IL-13 levels in the CRS group were decreased compared with those in the control group (Figure 2(c)). In short, CRS facilitated the inflammatory response in the endometrium of early pregnancy sows.

CRS stimulated NE production, and *β*_2_-adrenergic receptor (*β*_2_-AR) may be activated by CRS. In our study, CRS promoted the transcription (Figure 2(d)) and translation of *β*_2_-AR (Figure 2(e)). Therefore, the stress signal was transmitted into the cell and affected cellular signaling, which was involved in the KEAP1/NRF2 pathway [30]. In this study, the *KEAP1* mRNA in the CRS group was increased compared with that in the control group (Figure 2(d)). CRS inhibited the transcription of *NRF2* and its downstream genes, including *NQO1* and *HO-1* (Figure 2(d)). The *NRF2* and *HO-1* mRNA levels in the IS were increased compared with those in the NI (Figure 2(d)). Therefore, the decreased antioxidative ability may be related to KEAP1/NRF2. Meanwhile, the signal may be initiated by the *β*_2_-AR pathway. The AP-1 (C-JUN and C-FOS), the third messenger, also responds to OS. Our work showed that the *C-JUN* and *C-FOS* mRNA levels in the CRS group were increased compared with those in the control group (Figure 2(d)). In the CRS group, the *C-FOS* mRNA in the IS was increased compared with the NI group (Figure 2(d)). Overall, CRS promoted the NE binding to *β*_2_-AR and then affected the AP-1 to respond to OS.

The FOXO family is involved in the OS and inflammation [31]. In the IS and NI, the *FOXO1*, *FOXO3*, and *FOXO4* mRNA levels in CRS were increased compared with those in the control (Figure 2(d)). Meanwhile, CRS promoted the translation of FOXO1 in the IS and NI, and implantation facilitated further the translation of FOXO1 (Figure 2(e)) in the CRS group. To a large extent, the FOXO family responds only to CRS and then induces inflammation and decreases antioxidative ability. FOXO1, as the key regulatory factor, should be located in the endometrium to study the mechanism. In the endometrial luminal epithelium (ELE), the IOD of FOXO1 in CRS was increased compared with that in the control. In the control, the IOD of FOXO1 in the IS was decreased compared with that in the NI. In endometrial glandular epithelium (EGE), the IOD of FOXO1 in CRS was increased compared with that in the control (Figure 2(f)). Together, FOXO1 in the EGE and ELE were involved in CRS during early pregnancy. On the other hand, NF-*κ*B is a recognized inflammation-inducing factor. In our study, CRS promoted the transcription and translation of NF-*κ*B p65 (Figures 2(d) and 2(e)). In CRS, p-NF-*κ*B p65 protein in IS was increased compared with the NI (Figure 2(e)).

In brief, CRS triggered *β*_2_-AR to activate the AP-1 and then disturbed the KEAP1/NRF2 pathway, which blocked the antioxidative ability of the endometrium. Furthermore, the OS induced inflammation via the FOXO1 and NF-*κ*B pathways, especially in endometrial epithelial cells.

### 3.3. CRS Triggered Apoptosis in the Endometrial Epithelium

**Figure 3:**
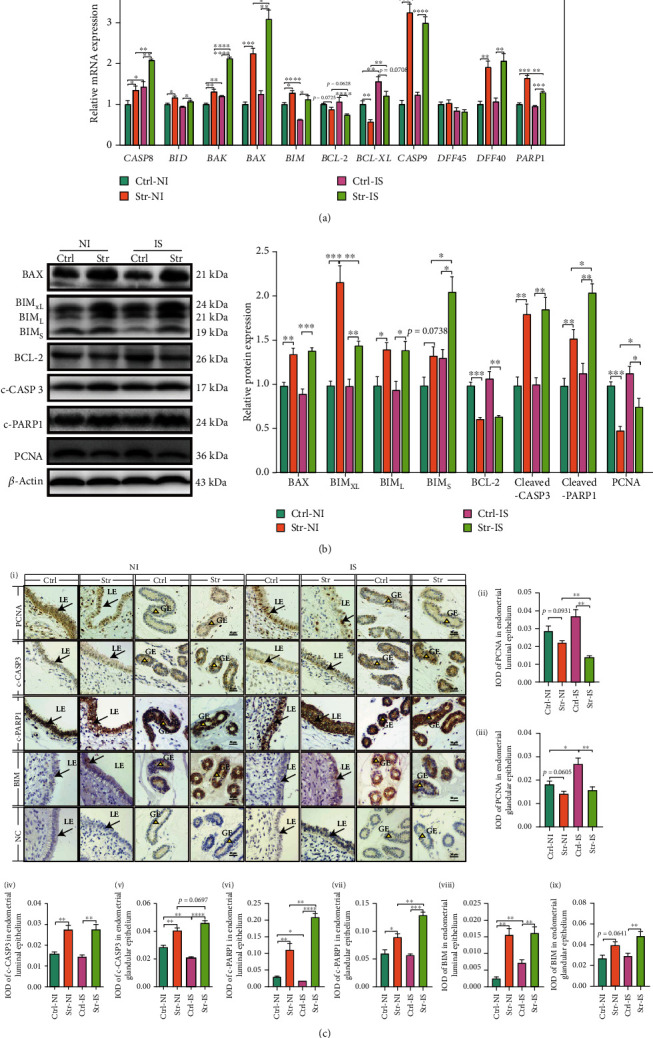
CRS triggered the apoptosis in the endometrial epithelium cells. (a) qRT-PCR was performed to measure the mRNA levels of proapoptosis genes (*CASP8*, *BID*, *BAX*, *BAK*, *BIM*, *CASP9*), antiapoptosis genes (*BCL-2* and *BCL-XL*) and apoptosis substrate genes (*DFF45*, *DFF40*, and *PARP1*). (b) Immunoblot analysis of proapoptosis protein (BAX, BIM, cleaved-CASP3, and cleaved-PARP1), anti-apoptosis protein (BCL-2), and proliferation protein (PCNA) in endometrium. *β*-Actin served as internal reference. (c) The representative images of Immunohistochemical staining (i) and semiquantitative of proliferation protein (anti-PCNA (ii and iii)), proapoptosis protein (anti-cleaved-CASP3 (iv and v) and anti-BIM (viii and ix)), and apoptosis substrate (anti-cleaved-PARP1 (vi and vii)). Apoptosis main happened in the epithelium. Meanwhile, CRS induced the apoptosis in endometrial epithelium cells. Bar: 50 *μ*m. IOD: integral optical density; NC: negative control. IS: implantation site; NI: nonimplantation site; Str: stress; Ctrl: control; *N* = 4, ^∗^*p* < 0.05, ^∗∗^*p* < 0.01, ^∗∗∗^*p* < 0.001, ^∗∗∗∗^*p* < 0.0001. ns: no significant.

From the above, CRS induced OS and inflammation and blocked endometrial development. Furthermore, the mechanism was explored. In our study, CRS initiated the transcription of *CASP8*, which acted on the *BID* to promote mitochondria to release the *BAX* and *BAK*. Increased *CASP9* promoted formation of apoptosis. Meanwhile, CRS stimulated the transcription of *BIM* to prevent the transcription of *BCL-2* and *BCL-XL* (Figure 3(a)). Normally, during pregnancy, apoptosis may be initiated due to the particular environment. However, this phenomenon only triggered the transcription of *CASP8* to increase the release of *BAK* and *BAX*. The transcription of *BIM* was decreased during implantation and could not inhibit the *BCL-2* and *BCL-XL* mRNA levels. During implantation, the endometrium was more sensitive to CRS, which caused an increase in the *CASP8*, *BAK*, and *BAX* and a decrease in the *BCL-2*. The apoptosis substrate was activated after CRS. In this study, CRS induced the transcription of *DFF40* but not *DFF45*, which implied DNA fragmentation. Meanwhile, the *PARP1*, as the substrate of CASP3, accumulated after CRS and was then cleaved by CASP3 (Figure 3(a)).

After CRS, the translation levels of BAX and BIM were facilitated to induce apoptosis and prevent antiapoptosis and proliferation (PCNA). As the apoptotic executor, cleaved CASP3 was increased by CRS and cleaved PARP1, which in turn produced cleaved PARP1. In contrast to transcription of PARP1, translation of cleaved PARP1 showed more intense expression in the IS under CRS (Figure 3(b)). Therefore, CRS triggered the caspase-dependent pathway to induce cell apoptosis. Moreover, in the implantation, proapoptosis and antiapoptosis were more influenced after CRS.

The apoptosis induced by CRS disturbed the development of the endometrium. However, the location of apoptosis is unclear. The IHC staining results showed that apoptosis occurred on ELE and EGE cells. In ELE, CRS increased the IOD of cleaved CASP3, cleaved PARP1, and BIM and decreased the IOD of PCNA. During implantation, the BIM was increased, but it did not cause PARP1 to be cleaved. In contrast, CRS made the ELE sensitive to apoptosis, which was implied through the increase in cleaved PARP1. Meanwhile, CRS decreased the proliferation ability in the IS. In the EGE, CRS elevated the IOD of cleaved CASP3, cleaved PARP1, and BIM and decreased the IOD of PCNA. In the control, PCNA was increased in the IS, and cleaved CASP3 was decreased in the IS of EGE. However, the IS was sensitive in CRS, which made the EGE more sensitive to cleaved CASP3 and cleaved PARP1 (Figure 3(c)). Overall, regardless of ELE or EGE, CRS induced apoptosis and was serious in the IS.

### 3.4. CRS Induced Autophagy in Endometrium Epithelium

**Figure 4:**
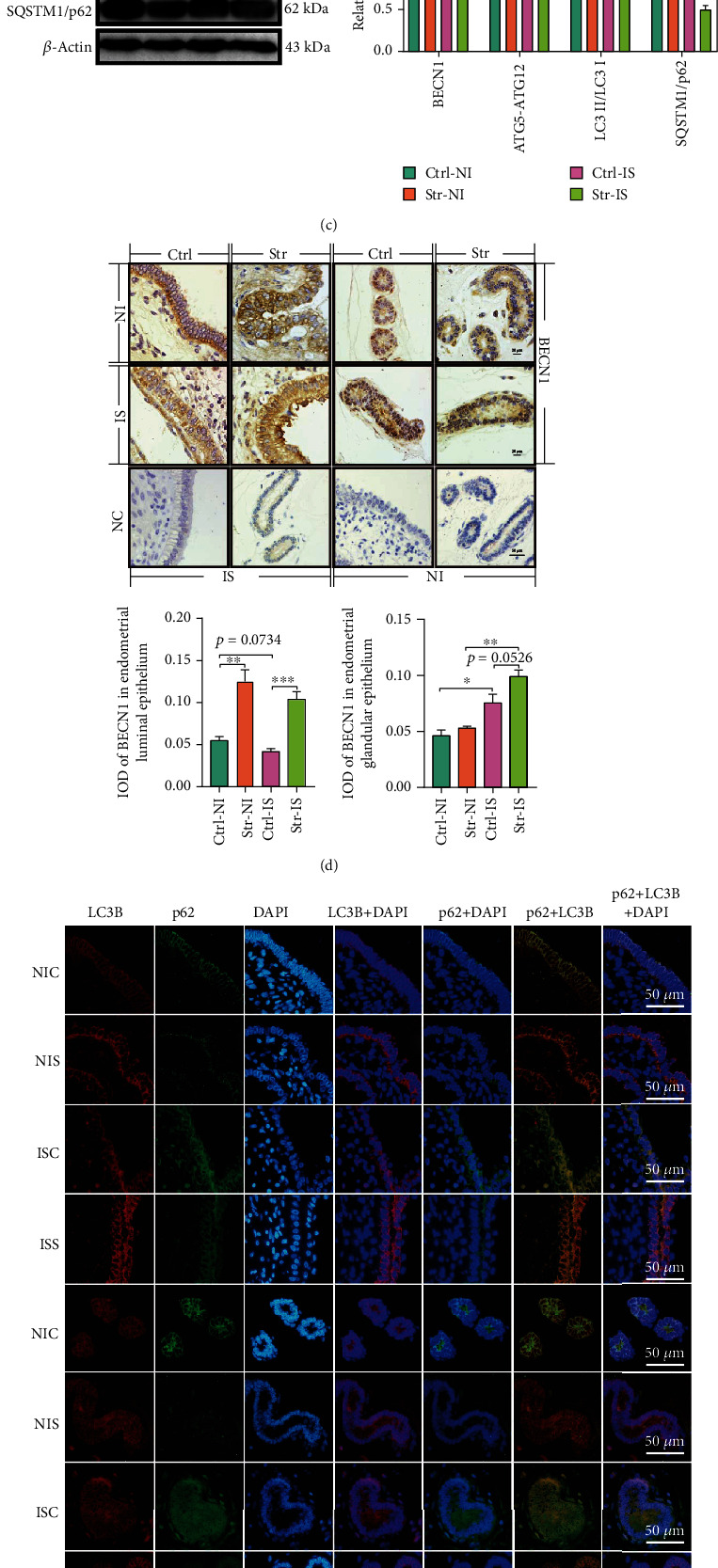
CRS induced the autophagy in endometrial epithelium cells. (a) qRT-PCR was performed to measure the autophagy-related genes (*ATG3*, *ATG5*, *ATG7*, *ATG12*, and *BECN1*) in endometrium. (b) The TEM image of luminal epithelium (LE) and glandular epithelium (GE). After CRS, the autophagosome (Avi) and autophagolysosome (Avd) (the black arrow) appeared. (c) Immunoblot analysis of MAP1LC3B-II accumulation, SQSTM1/p62 degradation, BECN1, and formation of ATG5-ATG12 complex. *β*-Actin served as the internal reference. (d) The representative images of immunohistochemical staining and semiquantitative analysis of BECN1 in LE and GE of uterine. Bar: 50 *μ*m. (e) Immunofluorescence microscopy was performed to visualize the MAP1LC3B (red) and SQSTM1/p62 (green) in the LE (first four rows) and GE (last four rows) of endometrium and semiquantitative analysis of MAP1LC3B and SQSTM1/p62 in epithelium. The nuclei were counterstained with DAPI (Blue). Bar: 50 *μ*m. Therefore, the autophagy main happened in the LE and GE after CRS. TEM: transmission electron microscope; DAPI: 4′,6-diamidino-2-phenylindole; IOD: integral optical density; MFI: mean fluorescence intensity; IS: implantation site; NI: nonimplantation site; Str: stress; Ctrl: control. *N* = 4, ^∗^*p* < 0.05, ^∗∗^*p* < 0.01, ^∗∗∗^*p* < 0.001, ^∗∗∗∗^*p* < 0.0001. ns: no significant.

In general, the OS can trigger autophagy to protect cells. Therefore, we explored the autophagy in the work. Our results demonstrated that the CRS initiated the transcription of *BECN1* to trigger autophagy-related genes, including the *ATG3*, *ATG5*, *ATG7*, and *ATG12*, to promote the formation of autophagic vacuoles and recruit the LC3 I to form LC3 II, which is a marker of autophagy. During implantation, the *ATG5* and *ATG12* were increased for successful pregnancy. However, *ATG7* was sensitive to CRS in the IS (Figure 4(a)). Transmission electron microscopy (TEM) was performed to observe autophagy. As shown in Figure 4(b), CRS induced the formation of autophagosomes and autophagolysosomes. Meanwhile, CRS initiated translation of BECN1 to trigger the ATG5-ATG12 complex protein, which promoted the accumulation of MAPILC3II and then increased SQSTM1/p62 degradation. Also, CRS increased BECN1 protein and promoted SQSTM1/p62 degradation during implantation (Figure 4(c)).

CRS initiated BECN1 in the ELE and EGE. In the EGE, the BECN1 was more easily initiated in the IS in the control group than in the NI, and CRS induced the initiation of BECN1 in the IS but not in the NI (Figure 4(d)). From Figure 4(e), we found that MAP1LC3B and SQSTM1/p62 colocalized on the ELE and EGE. In the ELE, CRS increased the mean fluorescence intensity (MFI) of MAP1LC3B and decreased SQSTM1/p62 (Figure 3(a)) (Figure 4(e)). After CRS, the MFI of MAP1LC3B showed intensive aggregation in the IS but not in the NI. In the IS, SQSTM/p62 degradation was increased compared with NI in CRS. In the EGE, CRS only elevated the MFI of MAP1LC3B in the IS but not in the NI and promoted SQSTM1/p62 degradation. In the IS, the changes in MAP1LC3B and SQSTM1/p62 were the same as those in the ELE. In CRS, the MFI of LC3B in the IS was increased, and the SQSTM1/p62 in the IS was increased compared with the NI (Figure 4(e)). Therefore, CRS can induce autophagy in the ELE and EGE. Moreover, the IS was sensitive to CRS-induced autophagy.

### 3.5. Duration and Intensity of H_2_O_2_ Affect Apoptosis and Autophagy in PEECs

*In vivo*, CRS induced apoptosis and autophagy in the ELE and EGE cells. To test the relative autophagy and apoptosis in the PEECs, we isolated the PEECs and performed identification with a CK19 antibody (Figure S1A). From the above *in vitro* results, it was known that CRS triggered the OS in endometrial epithelial cells. Therefore, the mechanism of autophagy and apoptosis was explored by adding H_2_O_2_ to the PEECs. The appropriate concentration of H_2_O_2_ was chosen via treatment with PEECs (Figure S1D and E). The H_2_O_2_ (50 *μ*M) caused the cell activity to decrease significantly after 3 h of treatment, and 200 *μ*M H_2_O_2_ began to cause a significant decrease in cell activity after 1 h (Figure S1C). To avoid the mechanistic disorder caused by the simultaneous occurrence of cell autophagy and apoptosis, we selected three groups of 50 *μ*M for 1 h and 3 h and 200 *μ*M for 1 h for subsequent experiments.

**Figure 5:**
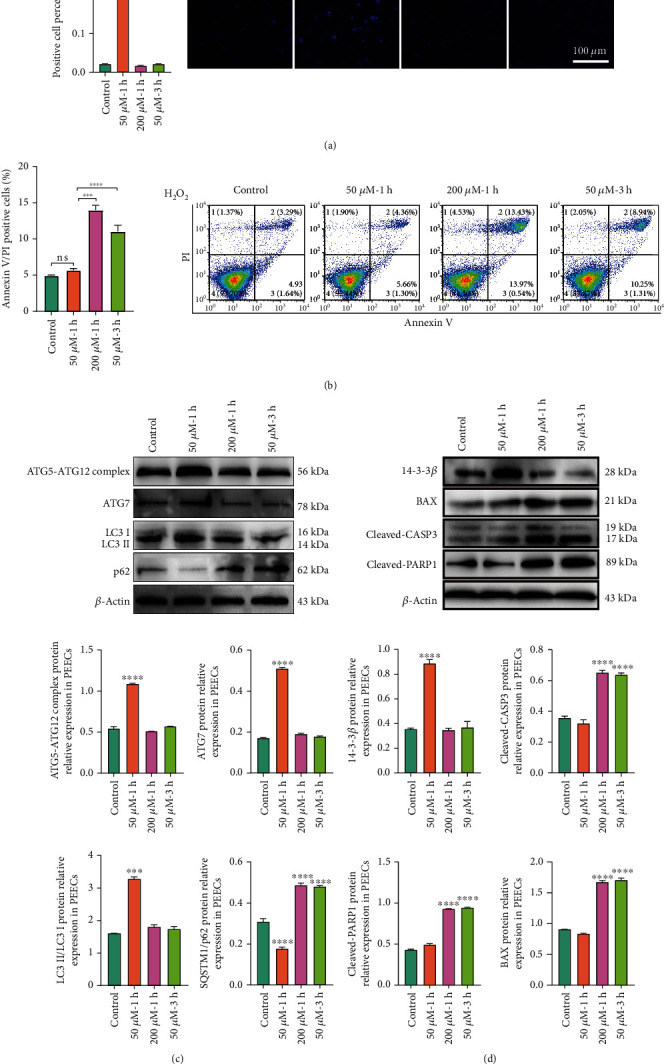
Duration and intensity of H_2_O_2_ affected apoptosis and autophagy in porcine endometrial epithelium cells (PEECs). (a) Dansylcadaverine (MDC) staining to verify the autophagosome cell after H_2_O_2_ treatment. Quantification of positive cells percent of MDC in H_2_O_2_ different treatment (50 *μ*M H_2_O_2_ for 1 h and 3 h or 200 *μ*M for 1 h). Bar: 100 *μ*m. (b) Flow cytometry was used to detect apoptosis after adding the 50 *μ*M H_2_O_2_ for 1 h and 3 h or 200 *μ*M for 1 h, apoptosis cells were measured. (c) The expression of ATG5-ATG12 complex, MAP1LC3B, SQSTM1/p62, and ATG7 was determined by Western blotting. The quantification of ATG5-ATG12 complex formation, ATG7 activation, MAP1LC3B accumulation, and SQSTM1/p62 degradation after H_2_O_2_ different durations and intensities in PEECs (50 *μ*M H_2_O_2_ for 1 h and 3 h or 200 *μ*M for 1 h). (d) Immunoblot analysis of 14-3-3*β*, BAX, cleaved-PARP1, and cleaved CASP3 in PEECs after H_2_O_2_ treatment (50 *μ*M H_2_O_2_ for 1 h and 3 h or 200 *μ*M for 1 h). *N* = 3, ^∗^*p* < 0.05, ^∗∗^*p* < 0.01, ^∗∗∗^*p* < 0.001, ^∗∗∗∗^*p* < 0.0001. ns: no significant.

The MDC staining showed that the percentage of MDCs was increased at 50 *μ*M-1 h but not at 200 *μ*M-1 h or 50 *μ*M-3 h (Figure 5(a)). Flow cytometry was used to detect apoptotic cells after adding 50 *μ*M H_2_O_2_ for 1 h and 3 h or 200 *μ*M H_2_O_2_ for 1 h. We found that the number of apoptotic cells in the 200 *μ*M-1 h and 50 *μ*M-3 h groups was increased compared with that in the 50 *μ*M-1 h group (Figure 5(b)). However, 50 *μ*M-1 h was not significantly different from the control. We speculated that the cell may trigger a protective mechanism to promote cell survival. Therefore, the different degrees of H_2_O_2_ may affect cell fate, which depends on the duration and intensification of OS. As a cell protection mechanism, autophagy is widespread in cells. Low OS promoted the formation of the ATG5-ATG12 complex and the expression of ATG7 to recruit MAP1LC3II (Figure 5(c)). To obtain a better assessment of the cells, the immunoblotting was performed to measure SQSTM1/p62 accumulation and degradation. Our work demonstrated that the low OS caused MAP1LC3II accumulation and the degradation of SQSTM1/p62. Meanwhile, SQSTM1/p62 was increased at 200 *μ*M-1 h and 50 *μ*M-3 h (Figure 5(c)), which was related to apoptosis initiation. Western blotting results demonstrated that low OS (50 *μ*M-1 h) induced the translation of 14-3-3*β* (Figure 5(d)), which can respond to the PI3K-AKT pathway to promote cell survival. However, high OS cannot active 14-3-3*β*. OS promoted mitochondria to release BAX in the 200 *μ*M-1 h and 50 *μ*M-3 h groups. Cleaved CASP3, the apoptotic executor, was induced by high OS. Furthermore, the CASP3 cleaved PARP1 in the 200 *μ*M-1 h and 50 *μ*M-3 h groups (Figure 5(d)). Therefore, high OS initiated the apoptosis. Together, the low OS induces autophagy to promote the PEECs survival, while the high OS triggers apoptosis to induce the PEEC death.

### 3.6. The Subcellular Localization of FOXO1 Affected the Occurrence of Autophagy and Apoptosis under OS

**Figure 6:**
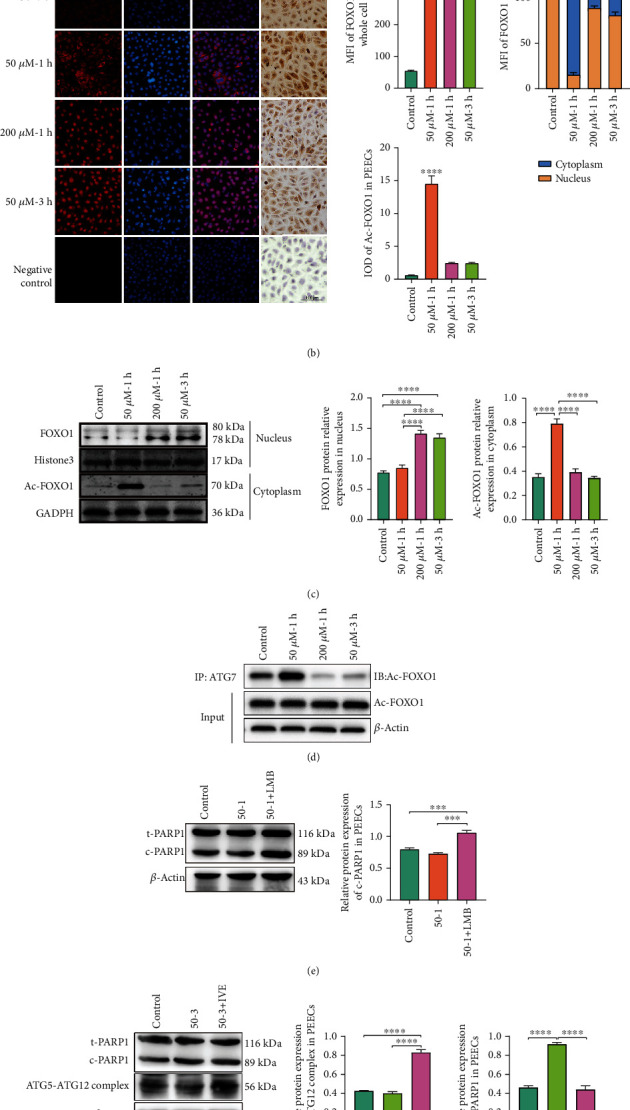
The cellular sublocalization of FOXO1 affected the occurrence of autophagy and apoptosis under OS. (a)The amino acid sequence showed NLS (nuclear localization sequence) and NES (nuclear export sequence) of FOXO1 (Sus scrofa). (b) Subcellular localization of FOXO1 in response to H_2_O_2_ different durations and intensities in PEECs using anti-FOXO1 (red), and the nuclei were counterstained via DAPI. Bar: 100 *μ*m. Meanwhile, the immunohistochemistry of Ac-FOXO1 to verify whether cause autophagy in low H_2_O_2_ (50 *μ*M H_2_O_2_ for 1 h) to protect cell. Bar: 100 *μ*m. The MFI of FOXO1 in whole cell to identify whether the change of FOXO1 in H_2_O_2_ different durations and intensities in PEECs. The MFI of FOXO1 in the nuclei (orange) and in the cytoplasm (blue). (c) Immunoblot analysis of nuclei separation to determine the FOXO1 in nucleus and Ac-FOXO1 in cytoplasm with H_2_O_2_ in different durations and intensities in PEECs (50 *μ*M H_2_O_2_ for 1 h and 3 h or 200 *μ*M for 1 h). Histone 3 served as the reference in nuclei and the GAPDH served as the reference in cytoplasm. (d) Representative images of Co-IP. (e) Immunoblot analysis of cleaved-PARP1 when the PEECs was treated with LMB before low OS. (f) Immunoblot analysis of cleaved-PARP1 and ATG5-ATG12 complex protein when the PEECs were treated with IVE before high OS. FOXO1 promoted autophagy in cytosol and facilitate apoptosis in nucleus. MFI: mean fluorescence intensity; *N* = 3, ^∗^*p* < 0.05, ^∗∗^*p* < 0.01, ^∗∗∗^*p* < 0.001, ^∗∗∗∗^*p* < 0.0001. ns: no significant.

The FOXO1 is the redox senor [21]. However, the effect of posttranslational modification of FOXO1 on cell fate under the OS is unclear. The protein sequence of FOXO1 was analyzed, and the NLS and NES regions were found at 252 aa to 281 aa and 378 aa to 387 aa (Figure 6(a)). Therefore, we performed an immunofluorescence assay. We found that the MFI of FOXO1 was enhanced at 50 *μ*M-1 h, 200 *μ*M-1 h and 50 *μ*M-3 h, which is consistent with the translation of FOXO1 *in vivo*. However, the FOXO1 in the nucleus was increased in 200 *μ*M-1 h and 50 *μ*M-3 h compared with 50 *μ*M-1 h. Of note, the FOXO1 was transferred into the cytoplasm in 50 *μ*M-1 h compared with 200 *μ*M-1 h and 50 *μ*M-3 h. Moreover, Ac-FOXO1 was increased in 50 *μ*M-1 h compared with the control but not in 200 *μ*M-1 h and 50 *μ*M-3 h (Figure 6(b)). Furthermore, we examined the translation of FOXO1 in the nucleus and Ac-FOXO1 in the cytoplasm. Our study suggested that the low OS induced the expression of Ac-FOXO1 in the cytoplasm. However, the high OS transmitted FOXO1 into the nucleus (Figure 6(c)). The ATG7 binds to Ac-FOXO1 in the cytoplasm when autophagy occurs. Here, the Co-IP assay showed that the low OS induced the binding of ATG7 and Ac-FOXO1 but not high OS (Figure 6(d)). The NLS and NES were blocked when the IVE and LMB were added. The obtained results demonstrated that cleaved PARP1 was increased when FOXO1 located in the nucleus (LMB+50 *μ*M-1 h group) under the low OS (Figure 6(e)). However, cleaved PARP1 was decreased and ATG5-ATG12 complex protein levels were increased when FOXO1 located in the cytoplasm (50 *μ*M-3 h + IVE) under high OS (Figure 6(f)). Therefore, autophagy occurs before apoptosis in PEECs under the OS. The shuttling of FOXO1 between the nucleus and cytoplasm determined the fate of the cell. The FOXO1 promoted transcription of proapoptotic genes to induce apoptosis in the nucleus, while the Ac-FOXO1 bound to ATG7 to trigger autophagy for cell survival.

## 4. Discussion

The World Health Organization (WHO) data showed that the conditions that occur during pregnancy and childbirth are one of the reasons for human death [32]. Moreover, we reviewed more articles and found that pregnancy diseases were related to oxidative stress [33]. Therefore, the mechanism of OS in reproductive disease should be explored.

In this study, the endometrium was studied systematically in early pregnancy sows under the SIS. Based on our data from *in vivo* models, the SIS induced the stress response. Stress activates the HPA axes and the sympathetic-adrenal medullary system (SAS), which induce the release of glucocorticoids from the adrenal cortex and the catecholamines epinephrine (EPI) and the NE from the adrenal medulla and sympathetic nerve termini [34]. In addition, gluconeogenesis is usually enhanced by glucocorticoids and then increases blood glucose levels [35]. The data in the study demonstrated that CRS triggered blood glucose, the COR and NE in plasma to activate the HPA axes, increased blood glucose levels, and affected the P4 and E2 in plasma, which was required for pregnancy. Evidence has established gestation as a period of vulnerability to environmental insults [36]. Stress impacts the HPO axes at the level of the hypothalamus and the pituitary gland, which exerts an effect on the ovary [37]. Circulating progesterone during early pregnancy in humans and other mammals is inversely correlated with circulating glucocorticoids [38], and stress exposure during pregnancy is associated with lower circulating progesterone concentrations [39, 40]. Stress can produce these adverse outcomes by increasing the activity of the HPA axes; when animals experience stress, the HPA axes releases glucocorticoids from the adrenal gland, and it (above homeostatic levels) impacts pregnancy progression and fetal development [26, 41].

The development of the uterus and endometrium gland was blocked due to the P4 and E2 disorders. The VEGF promotes angiogenesis by driving widespread events in the involved endothelial cells [42], which is beneficial for the development of the endometrium. The formation of blood vessels in the uterus is also hindered in pregnancy. The P4 receptor and E2 receptor signals were attenuated, which was beneficial for anti-implantation signals. Collectively, the CRS reduced the ability of uterine pregnancy, which was related to uterine endometrial cell fate.

CRS can increase the intracellular accumulation of ROS [43]. In accordance with this, the obtained results also showed that the SIS induced OS levels and extended the antioxidative ability, whether in plasma or in the endometrium. Moreover, CRS initiated KEAP1 binding to NRF2 to inhibit the transcription of downstream antioxidative factors. Thus, our results indicated that the key factor disturbing the development of the uterus was OS. Accumulating evidence has demonstrated that CRS exposure activates OS to trigger the release of inflammatory signaling molecules [8, 44, 45], which accelerate ageing and some chronic diseases [46]. There is evidence showing that OS is associated with decreased the GSH levels and increased the AP-1 and NF-*κ*B activation, leading to enhanced proinflammation in human alveolar epithelial cells (A549) [47]. Sika deer antler protein (SDAPR), SDAP1, and SDAP2 protect against APAP-induced OS and apoptosis by initiating NRF2 and restraining FOXO1 through the PI3K/AKT signaling [48].

The FOXO1, as a transcriptional regulator, is involved in cell proliferation, apoptosis, autophagy, OS, and metabolic dysregulation [49]. With a broad presence, the FOXO1 is demonstrated to be a representative member of the FOXO family, with key regulatory activities in transcription [50]. FOXO1 expression increased in the liver of mice after chronic stress, and FOXO1 upregulated the IL-1*β*, IL-6, TNF-*α*, and NF-*κ*B to promote inflammation [31]. Dioscin inhibited Dox-induced apoptosis and inflammation by inhibiting the nuclear translocation of FOXO1 and NF-*κ*B p65 [18]. Our results showed that CRS attenuates the anti-inflammatory ability and increases the proinflammatory ability via the AP-1/NF-*κ*B or FOXO1/NF-*κ*B pathways. Upstream of these signals is *β*_2_-AR, a G protein-coupled receptor, which is initiated by the NE in the endometrium. Moreover, these processes occurred in the ELE cells and the EGE cells in our current results.

ROS transmits signals as important second messengers and serves important regulatory functions in cell growth and differentiation to maintain cellular homeostasis at very moderate concentrations [51]. However, excessive ROS-induced OS impairs vital cell components, leading to cell cycle arrest and eventual apoptosis or necrosis [52, 53]. Similarly, in our study, the development of the uterus was related to apoptosis induced by the OS. In this study, we found that CRS initiated the BIM to inhibit the BCL-2 and BCL-XL and triggered the CASP8 to activate BID, accelerating mitochondrial release of BAX and BAK and further promoting CASP9 to form an apoptotic body to cleave CASP3, which cleaves PARP1. Finally, apoptosis occurs in cells. In the uterus, apoptosis occurs in epithelial cells in the endometrium.

Under some conditions, autophagy has a protective effect, preventing cells from undergoing apoptosis by promoting cell survival. Meanwhile, there is evidence that altered autophagy contributes to cell survival or cell death upon stress stimuli [54]. CRS suppresses adult hippocampal neurogenesis in mice by inducing autophagic cell death (ACD) of hippocampal neural stem cells (NSCs) [55]. Autophagic clearance of diverse damaged molecules may serve as an essential cellular antioxidant pathway [56]. It remains inconclusive whether autophagy can cause cell death. Our study demonstrated that CRS triggered autophagy and activated ATG genes to form autophagic vesicles to parcel cargo. Meanwhile, autophagosome formation was dependent on MAP1LC3-II accumulation. Finally, the SQSTM1/p62 degradation caused the autophagolysosome to release amino acids to be reused by cells. The process was beneficial for cell survival. Therefore, we suspected that CRS caused autophagy and apoptosis. However, the sequence was unclear.

Oxidative stress is a double-edged sword. A low degree of hydrogen peroxide is beneficial for the body. However, long-term exposure can cause injury to the body. Increasing evidence has demonstrated that the order of autophagy and apoptosis may be related to the duration and intensity of stress [57]. CHO induced apoptosis and autophagy simultaneously in tendon-derived stem cells (TDSCs) [19]. In addition to apoptosis, autophagy is another key factor controlling cellular fate. Numerous studies have revealed that autophagy plays a dual role, leading to cell death or promoting cell survival [58]. There is evidence that stress, particularly CRS, can reduce the pain threshold and increase pain sensitivity [59]. The consequences of stress depend on characteristics of the stressor, including the duration of exposure, severity, and predictability [60]. One speculation is that BH3-only proteins induce autophagy at low, initial levels of stress by activating the BECN1-VPS34 complex, when mitochondria are still protected against lethal permeabilization. However, at a higher, more advanced level of stress, BH3-only proteins induce MOMP, thereby setting off the apoptotic cascade [61]. Thus, BIM exemplifies a BH3-only protein, induces apoptosis, and inhibits autophagy [62, 63]. Similarly, in our study, low H_2_O_2_ induced autophagy to promote cell survival, while high H_2_O_2_ attenuated cell viability by promoting the apoptosis pathway. Therefore, there was a switch to respond to the duration and intensity of OS.

In pancreatic cancer, the PI3K-AKT can promote the binding of p-FOXO1 with 14-3-3 proteins to block apoptosis [64]. Nuclear localization of FOXO1 actuates the declaration of downstream factors, including the expression of the proapoptosis- related proteins BAD, BAX, BIM, CASP8, and CASP3 and downregulates the expression of SOD-1 and BCL-2, thereby advancing apoptosis [65–67]. The molecular mechanism driving FOXO1 action upon OS mainly consists of its posttranslational modification and nuclear accumulation [68]. The subtle mechanisms of posttranscriptional modifications and the effect of FOXO1 remain elusive and even conflicting in the development of many diseases [49]. This study demonstrated that FOXO1 was located in the cytoplasm under low H_2_O_2_ conditions and that FOXO1 was located in the nucleus under high H_2_O_2_ conditions. FOXO1 triggers apoptosis under OS [69]. Acetylated FOXO1 binds to ATG7 to influence the autophagic process [70–72]. In our study, the subcellular localization of FOXO1 affected the occurrence of autophagy and apoptosis under different degrees of OS.

## 5. Conclusion

**Figure 7:**
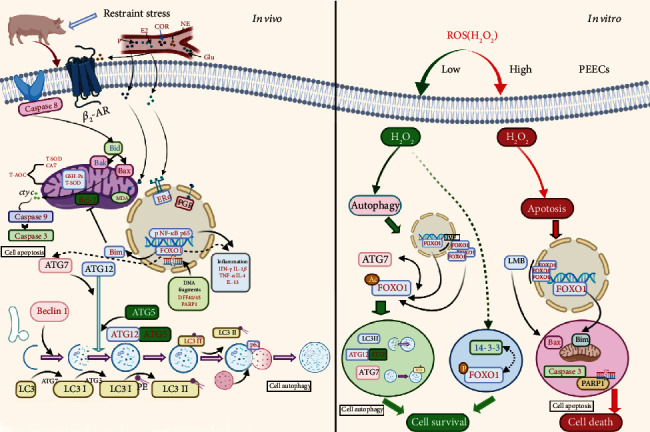
The summary of the study. *In vitro*, CRS induced the apoptosis and autophagy simultaneously; *in vivo*, autophagy precedes apoptosis, which was regulated by sublocalization of FOXO1.

Overall, this study emphasized the impact of stress intensity on the uterus of pregnant sows. CRS induced autophagy, apoptosis, and inflammation by initiating the OS and then blocked the development of endometrium in early pregnancy sows through the HPA and HPO axes. FOXO1, as a redox sensor switch, regulated the transformation of cell autophagy and apoptosis under different degrees of OS. The posttranslational modification of FOXO1 may become the target of OS treatment (Figure 7).

## Data Availability

The data that support the findings of this study are available from the corresponding author upon reasonable request.

## Abbreviations

A/G: Albumin/globulin
ACD: Autophagic cell death
ALP: Alkaline phosphatase
ALT: Alanine aminotransferase
AMY: Amylase
AST: Aspartate aminotransferase
BAND: Rod neutrophil percentage
BAS: Basophilic cell
BIM: B-cell lymphoma-2- (Bcl2-) like protein 1
CCK-8: Cell Counting Kit 8
CHO: Cholesterol
CK: Creatine kinase
COR: Cortisol
CRE: Creatinine
CRS: Chronic restraint stress
DBIL: Direct bilirubin
E2: Estradiol
EOS: Oxyphil cells
FOXOs: Forkhead box proteins
GE: Glandular epithelium
GGT: *γ*-Glutamyl transpeptidase
Glob: Globulin
ALB: Albumin
GLU: Glucose
HCT: Hematocrit
HGB: Hemoglobin
HPA: Hypothalamic-pituitary-adrenal
HPO: Hypothalamic-pituitary-ovary
HRP: Horseradish peroxidase
IVE: Ivermectin
LDH: Lactate dehydrogenase
LE: Luminal epithelium
LMB: Leptomycin B
LYM: Lymphocytes
MCH: Mean corpuscular hemoglobin
MCHC: Mean corpuscular hemoglobin concentration
MCV: Mean corpuscular volume
MFI: Mean fluorescence intensity
MOMP: Mitochondrial outer membrane permeabilization
MON: Monocytes
MST1: Mammalian sterile20-like kinase 1
NRF2: Nuclear factor-erythroid 2- (NF-E2-) related factor 2
NSCs: Neural stem cells
OS: Oxidative stress
P: Phosphorus
P4: Progesterone
PI: Propidium iodide
PLT: Platelet count
RBC: Red blood cell
REGA: Endometrium glandular area
ROS: Reactive oxygen species
SDAPR: Sika deer antler protein
SEG: Leafy neutrophil percentage
SIS: Sow individual stalls
TBA: Total bile acid
TBIL: Total bilirubin
TDSCs: Tendon-derived stem cells
TG: Triglyceride
TP: Total protein
UREA/CRE: Urea/creatinine
UREA: Urea
VEGF: Vascular endothelial growth factor
WBC: White blood cell.

## Additional Points

*Highlights*. (1) CRS induced oxidative stress in endometrium of early pregnancy sow. (2) CRS prevented development of endometrium. (3) CRS induced apoptosis and autophagy in endometrium of early pregnancy sow. (4) FOXO1 regulated the apoptosis and autophagy via subcellular localization

## Supplementary Materials

Supplementary Materials Supplementary data to this article can be found in Supplemental Files. The supplementary materials include two figures and three tables, Including identification and mycoplasma detection of PEECs, results of blood routine detection, the number of fetal, detection results of cell activity after adding hydrogen peroxide, and the results of data statistical analysis. Fig. S1: The routine blood test, identification of PEECs and the filter of H2O2 concentration and duration. Fig. S2: The number of fetal. Table S1: The list of primer sequence. Table S2: Antibodies used in this article and their concentration. Table S3: Table of statistical results of two-way ANOVA analyze.
