# Supplementary material for: FOXO1 Is a Critical Switch Molecule for Autophagy and Apoptosis of Sow Endometrial Epithelial Cells Caused by Oxidative Stress
Source: Oxid Med Cell Longev. 2021 Dec 21;2021:1172273. doi: 10.1155/2021/1172273 (PMC8714345; doi:10.1155/2021/1172273)
Supplement: Supplementary Materials — Supplementary data to this article can be found in Supplemental Files. The supplementary materials include two figures and three tables, Including identification and mycoplasma detection of PEECs, results of blood routine detection, the number of fetal, detection results of cell activity after adding hydrogen peroxide, and the results of data statistical analysis. Fig. S1: The routine blood test, identification of PEECs and the filter of H2O2 concentration and duration. Fig. S2: The number of fetal. Table S1: The list of primer sequence. Table S2: Antibodies used in this article and their concentration. Table S3: Table of statistical results of two-way ANOVA analyze. [file 1172273.f1.docx]

***Supplementary material***

**FOXO1 is a critical switch molecule for autophagy and apoptosis of sow endometrial epithelial cells caused by oxidative stress**

Jiayin Lu^1^, Jiaqiang Huang^2^, Shisu Zhao^1^, Wenjiao Xu^1^, Yaoxing Chen^1^, Yuanyuan Li^1^, Zixu Wang^1^, Yanjun Dong^1^, Renrong You^1^, Jing Cao^1^, Yulan Dong^1^

1. College of Veterinary Medicine, China Agricultural University, Haidian, Beijing 100193, People’s Republic of China.
2. Key Laboratory of Precision Nutrition and Food Quality, Ministry of Education, China Agricultural University, Haidian, Beijing 100193, People’s Republic of China.

Jiayin Lu, [lujiayin2013@163.com](mailto:lujiayin2013@163.com);

Jiaqiang Huang, [bornhuang@foxmail.com](mailto:bornhuang@foxmail.com);

Shisu Zhao, [zhaoshisuzss@163.com](mailto:zhaoshisuzss@163.com);

Wenjiao Xu, [xwjvet@163.com](mailto:xwjvet@163.com);

Yaoxing Chen, [yxchen@cau.edu.cn](mailto:yxchen@cau.edu.cn);

Yuanyuan Li, [lyydyxzz@163.com](mailto:lyydyxzz@163.com);

Zixu Wang, [zxwang2007@163.com](mailto:zxwang2007@163.com);

Yanjun Dong, [yanjund@cau.edu.cn](mailto:yanjund@cau.edu.cn);

Renrong You, [Renrong919@163.com](mailto:Renrong919@163.com);

Jing Cao, [caojing315@126.com](mailto:caojing315@126.com);

Yulan Dong, [ylbcdong@cau.edu.cn](mailto:ylbcdong@cau.edu.cn).

The co-first author:

Jiayin Lu and Jiaqiang Huang

E-mail: [lujiayin2013@163.com](mailto:lujiayin2013@163.com) and [bornhuang@foxmail.com](mailto:bornhuang@foxmail.com).

The corresponding author:

Yulan Dong

E-mail: [ylbcdong@cau.edu.cn](mailto:ylbcdong@cau.edu.cn).

**Figure S1. The routine blood test, identification of PEECs and the filter of H_2_O_2_ concentration and duration.** (A) The routine blood test assay. (B) Image of the results of mycoplasma detection. M: marker; P: positive control; N:Negative control; T: Test sample. (C) The PEECs were identified by Immunofluorescence using the anti-CK19 and the BSA was used to the negative control. (D) and (E) PEECs were incubated with different (50 μM, 100 μM, 200 μM, 400 μM, 800 μM and 1000 μM) H_2_O_2_ concentration for 0.5 h, 1 h, 2 h, 3 h, 6 h, 12 h, 24 h. Cell viability was then determined using the CCK8 assay *, *p*<0.05, **, *p*<0.01, ***, *p*<0.001, ****, *p*<0.0001. ns, no significant. Student’s t-test, mean ± SEM. N = 3.


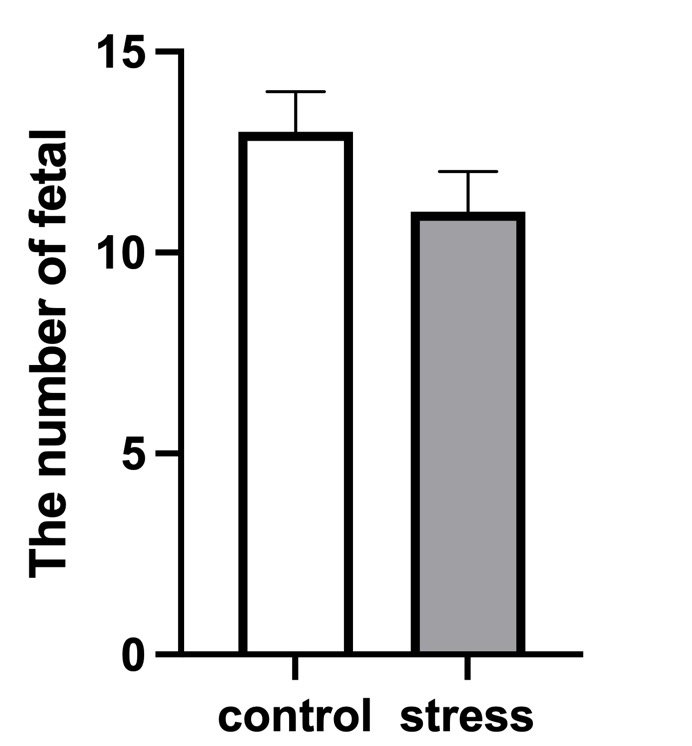


Figure S2. The number of fetal.

**Table S1. The list of primer sequence.**

| ***Gene name*** | **Forword sequences (5'-3')** | **Resever sequence (5'-3')** | **Product length** | | **Source** |
| --- | --- | --- | --- | --- | --- |
| *Bax* | TTCAGGGTTTCATCCAGGATCG | ATCCTCTGCAGCTCCATGTTAC | | 105 | XM_003127290.5 |
| *Bcl-2* | GATGCCTTTGTGGAGCTGTATG | CCCGTGGACTTCACTTATGG | | 144 | XM_021099593.1 |
| *BCL-xl* | GAGCAGGTATTGAACGAACTCTTCC | TTCCACAAAAGTGTCCCAGCC | | 209 | NM_214285.1 |
| *caspase 9* | ATTGGTTCTGGAGGATTCAGTG | GTTGTTGATGATGAGGCAGTG | | 113 | XM_003127618.4 |
| *caspase-8* | TGAGGAGGGCTGATTCCCTAT | TGGCTCTCCTCTTGCATACC | | 108 | NM_214131.1 |
| *BAK1* | GACATCAACCGGCGATACGA | CTGGAGGCGATCTTGGTGAA | | 101 | XM_003127618.4 |
| *Bimα1* | AGTGCGATGGCTTCCATGAG | AAGAAAACAGCATTACCCTCCT | | 135 | NM_001252194.1 |
| *BID* | GGGGACAGGATGGAGTATGG | GGTGAGGAGGTTCTGGTTGAT | | 273 | NM_001030535.1 |
| *Beclin1* | AGTAGGTGAAGGCTAGGCGAT | TGACACACGAAGCTCACCTG | | 167 | NM_001044530.1 |
| *ATG7* | CGGATGGTGAACCTCAGCGA | CATACAGCGGCTGCCTCACAG | | 261 | NM_001190285.1 |
| *ATG3* | CACGACTATGGTTGTTTGGCTATG | GGTGGAAGGTGAGGGTGATTT | | 126 | XM_003132682.6 |
| *ATG12* | CTACGGAGGTCTCCCCAGAA | AAATAAACAACTGTTCTGAAGCCA | | 243 | NM_001190282.1 |
| *ATG5* | GCGCTTCTGTTTGGAGTTGG | TGGCAAAAGCAAATAATACGGT | | 186 | NM_001037152.2 |
| *DFF45* | ATAAGTCCCTGGCACCCGTCAC | CCCGCTGTCTGTTTCATCTC | | 199 | XM_003127563.4 |
| *DFF40* | GGAACCTGGACCACATAATAGA | GGTGAAGAGCAGGCTGTAGAA | | 119 | XM_021095232.1 |
| *HO-1* | AGCTGTTTCTGAGCCTCCAA | CAAGACGGAAACACGAGACA | | 130 | NM_001004027.1 |
| *PARP1* | AGTATGCCAAGTCCAACAGA | CCATCTACCTCGTCACCTTT | | 298 | XM_003357641.4 |
| *Keap1* | AGCTGGGATGCCTCAGTGTT | AGGCAAGTTCTCCCAGACATTC | | 100 | NM_001114671.1 |
| *NQO1* | CCAGCAGCCCGGCCAATCTG | AGGTCCGACACGGCGACCTC | | 160 | NM_001159613.1 |
| *Nrf2* | GAAAGCCCAGTCTTCATTGC | TTGGAACCGTGCTAGTCTCA | | 190 | XM_003133500.6 |
| *FOXO1* | GCAAATCGAGTTACGGAGGC | AATGTCATTATGGGGAGGAGAGT | | 95 | NM_214014.3 |
| *FOXO3* | GGGGAGTTTGGTCAATCAGA | TGCATAGACTGGCTGACAGG | | 168 | XM_021084231.1 |
| *FOXO4* | CGCCAAGCCAAGATCGAATG | GCTCAGGGATCTGGCTCAAA | | 142 | XM_003135172.4 |
| *NF-κB P65* | AGTACCCTGAGGCTATAACTCGC | TCCGCAATGGAGGAGAAGTC | | 133 | NM_001114281.1 |
| *β_2_-AR* | TGCCTGCTGACCAAGAACAA | GACACGATGGAAGAGGCGAT | | 194 | NM_001128436.1 |
| *c-Jun* | TCCAGTAACGGGCACATCAC | CCCTCCTGCTCGTCAGTCAC | | 80 | NM_213880.1 |
| *c-Fos* | TCCCAACGGTGACTGCTATC | CCTCCTGGCATGGTCTTCAC | | 172 | NM_001123113.1 |
| *UBB* | GTCTGAGGGGTGGCTGCTAA | TGGGGCAAATGGCTAGAGTG | | 85 | XM_005653994.3 |

**Table S2. Antibodies used in this article and their concentration**

| Antibodies | manufacturer | Catalog numbers | Dilution (WB) | Dilution (IHC/ICC) | Dilution (IF) |
| --- | --- | --- | --- | --- | --- |
| SQSTM1/p62 | Cell signaling technology | 88588 | 1:1000 |  | 1：100 |
| LC3B | Novus | NB100-2220 | 1:1000 |  | 1：100 |
| Beclin1 | CUSABIO | CSB-PA617917LA01HU | 1:1000 | 1：100 |  |
| ATG7 | CUSABIO | CSB-PA002294LA01HU | 1:1000 |  |  |
| ATG5-ATG12 | Santa cruze | A0731 | 1:1000 |  |  |
| Bax | proteintech | 50599-2-Ig | 1:4000 |  |  |
| Bcl-2 | proteintech | 12789-1-AP | 1:1000 |  |  |
| Bim | Santa cruze | Sc-374358 | 1:1000 | 1：200 |  |
| Cleaved-Caspase3 | Cell signaling technology | 9661 | 1:1000 | 1：300 |  |
| Cleaved-PARP1 | Cell signaling technology | CSB-PA000080 | 1:1000 |  |  |
| PARP1 | CUSABIO | 5942 | 1:1000 | 1:100 |  |
| 14-3-3β | CUSABIO | CSB-PA0154410Rb | 1:1000 |  |  |
| FOXO1 | Cell signaling technology | 2880 | 1:1000 | 1:100 | 1:100 |
| Ac-FOXO1 | Affinity | AF2305 | 1:1000 | 1:100 |  |
| PGR | CUSABIO | CSB-PA003846 | 1:1000 |  |  |
| ERα | Bioss | bs-6998R | 1:1000 |  |  |
| Muc1 | Novus | NBP1-60046 | 1:1000 |  |  |
| β_2_-AR | abclonal | A1295 | 1:1000 |  |  |
| p-NF-κB p65 | abclonal | AP0124 | 1:1000 |  |  |
| PCNA | proteintech | 60097-1-Ig | 1:1000 | 1：500 |  |
| GADPH | CUSABIO | CSB-MA000071M0m | 1:2000 |  |  |
| Histone 3 | CUSABIO | CSB-PA01435A0Rb | 1:1000 |  |  |

| Name | Group | Site | Group*Site | Name | Group | Site | Group*Site |
| --- | --- | --- | --- | --- | --- | --- | --- |
| REGA | P<0.05 | P<0.05 | P>0.05 | BCL-XL mRNA | P<0.05 | P<0.05 | P>0.05 |
| VEGF | P<0.05 | P>0.05 | P<0.05 | CASP9 mRNA | P<0.05 | P>0.05 | P>0.05 |
| ERα protein | P<0.05 | P<0.05 | P<0.05 | DFF45 mRNA | P>0.05 | P<0.05 | P<0.05 |
| PGR protein | P<0.05 | P>0.05 | P>0.05 | DFF40 mRNA | P<0.05 | P>0.05 | P>0.05 |
| MUC1 protein | P<0.05 | P<0.05 | P>0.05 | PARP1 mRNA | P<0.05 | P<0.05 | P<0.05 |
| CAT | P<0.05 | P>0.05 | P>0.05 | BAX protein | P<0.05 | P>0.05 | P>0.05 |
| GSH-PX | P<0.05 | P<0.05 | P<0.05 | BIM_XL_ protein | P<0.05 | P<0.05 | P<0.05 |
| T-SOD | P<0.05 | P<0.05 | P>0.05 | BIM_L_ protein | P<0.05 | P>0.05 | P>0.05 |
| T-AOC | P<0.05 | P>0.05 | P<0.05 | BIM_S_ protein | P<0.05 | P<0.05 | P>0.05 |
| MDA | P<0.05 | P<0.05 | P>0.05 | BCL-2 protein | P<0.05 | P>0.05 | P>0.05 |
| IFN-γ | P<0.05 | P<0.05 | P<0.05 | C-CASP3 protein | P<0.05 | P>0.05 | P>0.05 |
| IL-1β | P<0.05 | P<0.05 | P<0.05 | C-PARP1 protein | P<0.05 | P<0.05 | P>0.05 |
| TNF-α | P<0.05 | P<0.05 | P>0.05 | PCNA protein | P<0.05 | P<0.05 | P>0.05 |
| IL-13 | P<0.05 | P>0.05 | P>0.05 | PCNA ELE | P<0.05 | P>0.05 | P<0.05 |
| IL-4 | P<0.05 | P<0.05 | P>0.05 | PCNA EGE | P<0.05 | P<0.05 | P>0.05 |
| C-JUN mRNA | P<0.05 | P>0.05 | P>0.05 | C-CASP3 ELE | P<0.05 | P>0.05 | P>0.05 |
| C-FOS mRNA | P<0.05 | P<0.05 | P>0.05 | C-CASP3 EGE | P<0.05 | P>0.05 | P<0.05 |
| KEAP1 mRNA | P<0.05 | P<0.05 | P>0.05 | PARP1 ELE | P<0.05 | P<0.05 | P<0.05 |
| NRF2 mRNA | P<0.05 | P<0.05 | P<0.05 | PARP1 EGE | P<0.05 | P<0.05 | P<0.05 |
| NQO1 mRNA | P>0.05 | P<0.05 | P>0.05 | BIM ELE | P<0.05 | P>0.05 | P>0.05 |
| HO-1 mRNA | P<0.05 | P<0.05 | P<0.05 | BIM EGE | P<0.05 | P>0.05 | P>0.05 |
| FOXO1 mRNA | P<0.05 | P>0.05 | P>0.05 | BECN1 mRNA | P<0.05 | P>0.05 | P>0.05 |
| FOXO3 mRNA | P<0.05 | P>0.05 | P>0.05 | ATG3 mRNA | P<0.05 | P<0.05 | P>0.05 |
| FOXO4 mRNA | P<0.05 | P<0.05 | P<0.05 | ATG5 mRNA | P<0.05 | P<0.05 | P>0.05 |
| NF-κB -p65 mRNA | P<0.05 | P<0.05 | P>0.05 | ATG7 mRNA | P<0.05 | P>0.05 | P<0.05 |
| β_2_-AR mRNA | P<0.05 | P>0.05 | P<0.05 | ATG12 mRNA | P<0.05 | P>0.05 | P<0.05 |
| FOXO1 protein | P<0.05 | P<0.05 | P>0.05 | BECN1 protein | P<0.05 | P<0.05 | P<0.05 |
| p-NF-κB p65 protein | P<0.05 | P<0.05 | P<0.05 | ATG5-ATG12 protein | P<0.05 | P>0.05 | P>0.05 |
| β_2_-AR protein | P<0.05 | P<0.05 | P>0.05 | LC3Ⅱ/Ⅰ | P<0.05 | P>0.05 | P>0.05 |
| FOXO1EGE | P<0.05 | P<0.05 | P<0.05 | P62 protein | P<0.05 | P<0.05 | P>0.05 |
| FOXO1ELE | P<0.05 | P<0.05 | P>0.05 | BECN1 ELE | P<0.05 | P>0.05 | P>0.05 |
| CASP8 mRNA | P<0.05 | P<0.05 | P>0.05 | BECN1 EGE | P<0.05 | P<0.05 | P>0.05 |
| BID mRNA | P<0.05 | P<0.05 | P>0.05 | LC3B ELE | P<0.05 | P<0.05 | P<0.05 |
| BAK mRNA | P<0.05 | P<0.05 | P<0.05 | LC3B EGE | P<0.05 | P<0.05 | P<0.05 |
| BAX mRNA | P<0.05 | P<0.05 | P>0.05 | SQSTM1/62 ELE | P<0.05 | P<0.05 | P<0.05 |
| BIM mRNA | P<0.05 | P<0.05 | P>0.05 | SQSTM1/p62 EGE | P<0.05 | P<0.05 | P<0.05 |
| BCL-2 mRNA | P<0.05 | P>0.05 | P>0.05 |  |  |  |  |

**Table S3.** **Table of statistical results of two-way ANOVA analyze.**

Note: Group: stress and control; Site: non-implantation site and implantation site. Group*Site: interaction between group and site. P <0.05: represents the corresponding indicators are greatly affected by this factor.
